# Cotransplantation of marginal mass allogeneic islets with 3D culture-derived adult human skin cells improves glycemia in diabetic mice

**DOI:** 10.1590/1414-431X2023e12611

**Published:** 2023-09-22

**Authors:** L. Andreone, A.F. dos Santos, R.A.M. Wailemann, L.F. Terra, V.M. Gomes, J. Macedo da Silva, L. Rosa-Fernandes, M.C. Sogayar, G. Palmisano, L. Labriola, M.J. Perone

**Affiliations:** 1 Immuno-Endocrinology, Diabetes & Metabolism Laboratory, Instituto de Investigaciones en Medicina Traslacional (IIMT), CONICET - Universidad Austral, Pilar, Argentina; 2 Facultad de Ciencias Biomédicas, Universidad Austral, Pilar, Argentina; 3 Departamento de Bioquímica, Instituto de Química, Universidade de São Paulo, São Paulo, SP, Brasil; 4 Departamento de Parasitologia, Instituto de Biosciências, Universidade de São Paulo, São Paulo, SP, Brasil; 5 Centro de Terapia Celular e Molecular, Faculdade de Medicina, Universidade de São Paulo, São Paulo, SP, Brasil

**Keywords:** Mesenchymal stem cells, Cell therapy, Type 1 diabetes, Three-dimensional (3D) culture, Hanging drop

## Abstract

Islet transplantation represents a therapeutic option for type 1 diabetes (T1D). Long-term viability of transplanted islets requires improvement. Mesenchymal stromal cells (MSCs) have been proposed as adjuvants for islet transplantation facilitating grafting and functionality. Stem cell aggregation provides physiological interactions between cells and enhances the *in situ* concentration of modulators of inflammation and immunity. We established a hanging-drop culture of adult human skin fibroblast-like cells as spheroids, and skin spheroid-derived cells (SphCs) were characterized. We assessed the potential of SphCs in improving islet functionality by cotransplantation with a marginal mass of allogeneic islets in an experimental diabetic mouse model and characterized the secretome of SphCs by mass spectrometry-based proteomics. SphCs were characterized as multipotent progenitors and their coculture with anti-CD3 stimulated mouse splenocytes decreased CD4+ T cell proliferation with skewed cytokine secretion through an increase in the Th2/Th1 ratio profile. SphCs-conditioned media attenuated apoptosis of islets induced by cytokine challenge *in vitro* and importantly, intratesticular SphCs administration did not show tumorigenicity in immune-deficient mice. Moreover, SphCs improved glycemic control when cotransplanted with a marginal mass of allogeneic islets in a diabetic mouse model without pharmacological immunosuppression. SphCs' protein secretome differed from its paired skin fibroblast-like counterpart in containing 70% of up- and downregulated proteins and biological processes that overall positively influenced islets such as cytoprotection, cellular stress, metabolism, and survival. In summary, SphCs improved the performance of transplanted allogeneic islets in an experimental T1D model, without pharmacological immunosuppression. Future research is warranted to identify SphCs-secreted factors responsible for islets' endurance.

## Introduction

Type 1 diabetes (T1D) is a progressive autoimmune disease characterized by T-cell-mediated dysfunction and loss of insulin-producing β cells. Lifelong exogenous insulin administration is required for patient survival, although it is only a palliative agent in T1D. Therapeutic interventions with immunosuppressive drugs have failed to achieve lasting remission, and beneficial effects have been observed only for short periods during treatment.

The use of cell therapy to repair damaged tissues, including islets and β cells in T1D (1) has attracted attention. Notably, the significant advance introduced by the use of potent induction therapy in addition to treatment with anti-cytokines before transplantation leads to 5-year insulin independence rates greater than 50%, as reported by the 2014 Collaborative Islet Transplant Registry (CITR) (2).

Stem cells have shown promising experimental results, but their use is limited because of the low survival rates and susceptibility to unfavorable stress conditions after transplantation due to ischemia, mechanical and oxidative stress, and inflammation, all of which contribute to cell loss. Multipotent stem cells, including mesenchymal stromal cells (MSCs), differentiate into cell types of a specific germ layer. Embryonic stem (ES) cells and induced pluripotent stem cells (iPSCs) have the potential to give rise to cells of all three germ lineages (e.g., mesodermal, endodermal, and ectodermal) (3). Concerns have arisen regarding the use of ES cells due to teratoma formation resulting from uncontrolled self-renewal and triploblastic differentiation. Although iPSC use has resolved the bioethical issues concerning embryonic human origin, the high rate of teratoma formation following transplantation has hindered their use in regenerative medicine.

MSCs can be easily isolated and expanded *in vitro* and have been employed in T1D clinical trials. Although studies have reported a modest preservation of residual β cells after MSCs infusion, prospective long-term studies showing consistent insulin independence are needed (4).

Distinctive pluripotent stem cells have been isolated from adult human tissues. Multilineage-differentiating stress-enduring (Muse) cells are stress-tolerant and express pluripotency markers. They are good candidates for treating inflammation as well as attenuating Th1 lymphocyte effector activity by downregulating the secretion of proinflammatory cytokines (e.g., IFN-γ and TNF-α) in an antigen-specific way *in vitro* (5). Infusion of Muse cells attenuated hyperglycemia in an experimental mouse model of T1D (6).

Allogeneic islet transplantation has emerged as a reliable therapeutic alternative for severe T1D. With islet transplantation, precise glycemic control over insulin administration should be achieved. However, it remains a restricted therapy due to a shortage of cadaveric donors and the lack of adequate immunosuppressive regimens (1). Very few transplanted patients achieve insulin independence for periods longer than one year. To improve islet functionality and promote insulin independence, the use of MSCs to provide a favorable niche for islet engraftment arose as an option to be tested in T1D models. Cotransplantation of MSCs and islets guarantees the provision of factors that improve the long-term islet viability. MSCs increase insulin secretion *in vitro* and improve transplantation outcomes. They modulate immune responses by secreting cytokines and soluble factors in the microenvironment. Immune modulation is a key factor in the context of pancreatic islet allotransplantation, and MSCs help to prevent host rejection while nourishing the graft (7).

MSCs grown as spheroids, i.e., 3D MSCs culture, have been shown to enhance the secretion of downregulators of the immune response (8). This finding suggests that this methodology could be used to improve islet allotransplantation in a diabetic mouse model.

In this study, we described that spontaneous formation of spheroids confers adult human skin fibroblast-like cell characteristics that improve marginal mass allogeneic islet transplantation in a diabetic mouse model.

The quantitative spheroid-derived cells (SphCs) secretome differs from that of its skin fibroblast-like counterparts in containing proteins with putative beneficial effects on islet function and survival. Further studies might determine whether these proteins sustain immunomodulation and cytoprotective effects in transplanted islets. This methodology could help improve transplant outcomes by reducing the undesirable consequences of pharmacological immunosuppression, as well as the number of islets required to achieve normoglycemia in T1D recipients.

## Material and Methods

### Mice

BALB/c, C57BL/6, and BALB/c nude mice (6-9 weeks old) were obtained from the animal care facility (Chemistry Institute; University of São Paulo). They were housed under a pathogen-free controlled environment (20-22°C, 12-h light/dark cycle) in ventilated cages and provided with food and water *ad libitum*. All procedures were conducted in accordance with the Guide for the Care and Use of Laboratory Animals, eighth edition (2011).

### Generation of spheroid-derived cells (SphCs) from adult skin fibroblast-like cells

Abdominal or mammary gland skin pieces were obtained from 3 females (29-46 years old). Skin pieces (5×5 mm) were treated with 10 µg/mL collagenase (Serva, Germany). Cells were cultured with DMEM 15% FBS, 2 mM L-glutamine, and 100 µg/mL streptomycin/ampicillin at 37°C/5% CO_2_ until 7-9 passages. Cells were plated as hanging drops on an inverted culture dish lid in 30 µL of DMEM+FBS at 5×10^4^ cells/drop for 72 h. Spheroids were transferred to adherent culture plates, and cells at passages 3-9 were used for experiments. FBS-free conditioned medium was harvested at 48 h.

### Surface phenotypic characterization of SphCs

Adult human skin cells and SphCs were harvested using trypsin-EDTA, fixed (2% PFA), and incubated with fluorochrome-conjugated antibodies: anti-CD105-APC, -CD90-FITC, -CD73-PE, -CD29-PE, -CD34-PE, -CD45-FITC, -HLA-DR-PE, and controls with isotype-matched irrelevant monoclonal antibodies: FITC-mIgG1, APC-mIgG1 and PE-mIgG2a (eBioLegend, USA). Analyses were performed by FACSVerse (BD Biosciences, USA) and FlowJo V10 software (BD Biosciences). At least 5×10^4^ events were acquired per sample (5).

### Evaluation of osteogenic, adipogenic, and chondrogenic differentiation of SphCs

SphCs were seeded at 70-80% confluence, cultured in tissue-specific induction medium, fixed, and stained, as described previously (5).

### Splenocyte assays

Mice splenocytes were obtained as described previously (5). Both primary skin fibroblast-like cells (10^4^) and SphCs (10^4^) were cocultured for 72 h with 5×10^5^ splenocytes activated with anti-CD3 antibody (1 mg/mL, eBioscience). A CellTrace Proliferation Kit (Thermo Fisher Scientific, USA) was used to evaluate T-CD4^+^ proliferation by staining with anti-CD4-APC. Cell media were harvested at 36 h, and cytokines were analyzed by FACS using a Cytometric Bead Array Mouse Th1/Th2/Th17 Cytokine Kit (BD Biosciences).

### Islet isolation and treatments

Pancreatic islets were isolated from BALB/c male mice. Islets were pretreated for 1 h with 20% conditioned medium from skin fibroblasts or SphCs, followed by exposure to a proinflammatory cytokine cocktail (8 ng/mL TNF-α, 4 ng/mL IFN-γ, and 0.8 ng/mL IL-1β; PeproTech, USA). After 24 h, islets were labeled with 1 μM Newport Green, 1.5 µM propidium iodide (PI), and 1 µg/mL Hoechst 33342. The death rate of each islet was considered the PI-positive area (nonviable cells), which was divided by the total area of the islet. Quantification was evaluated by ImageJ software (NIH, USA).

Five islets/well were cultured for 24 h with 20% conditioned medium from skin fibroblasts or SphCs. Islets were incubated with Krebs buffer (Sigma-Aldrich, USA) supplemented with 0.2% BSA and 5.6 mM glucose for 30 min at 37°C. The buffer was replaced with Krebs buffer containing 0.2% BSA/2.8 mM glucose for 1 h. The supernatant was collected, and islets were incubated for 1 h in Krebs buffer (0.2% BSA/16.7 mM glucose).

### Cotransplantation of marginal mass allogeneic islets with SphCs in diabetic mice

Diabetes was induced in C57BL/6 mice by streptozotocin (*ip*, 170 mg/kg body weight STZ). To establish the islet marginal mass, two different numbers of islet equivalents (IEQs) from BALB/c mice were transplanted under the kidney capsule of diabetic C57BL/6 mice (300 IEQ and 600 IEQ). An IEQ was an islet mass with a diameter of 125 µm.

Diabetic recipients of marginal mass islets were divided into 4 experimental groups as follows: sham (n=12), islets (300 IEQ; n=9), 300 IEQ cotransplanted with 1×10^4^ skin cells (parental; n=9), or 300 IEQ cotransplanted with 1×10^4^ SphCs (SphCs; n=10). Recipients were followed for 10 days to measure nonfasting blood glucose with a glucometer (Contour™TS, Bayer, Japan). Diabetes was considered after two consecutive blood glucose levels were ≥300 mg/dL.

### Tumorigenesis assay

Adult skin cells and SphCs were suspended (1×10^5^ in PBS) and intratesticularly (*it*) injected into BALB/c nude mice (5). P19 cells were injected (1×10^5^) as a positive control.

### Proteomic sample preparation

Proteins were concentrated using a 10 kDa filter (Millipore, USA). The concentrate was diluted to 8 M urea/50 mM NH_4_HCO_3_ and treated with 10 mM ditiotreitol (DTT) for 1 h at 30°C. Reduced cysteines were alkylated with 55 mM iodoacetamide/50 mM NH_4_HCO_3_ for 45 min before trypsin (Promega, USA) digestion at a 1:50 ratio. Tryptic peptides were desalted using Stage Tips with C18 disks (Sigma Aldrich).

### Nano LC-MS/MS analysis

Peptides were suspended in 0.1% formic acid (FA) before analysis using a nanoflow EASY-nLC™ II system (Thermo Scientific, USA) coupled to an LTQ-Orbitrap Velos mass spectrometer (Thermo Scientific). Then, the samples were loaded on an Acclaim PepMap C18 (Thermo, Germany) trap column (2 cm × 100 µm; inner diameter 5 µm) and separated onto an Acclaim PepMap C18 (15 cm × 75 µm; inner diameter 3 µm) column. A 70-min gradient was used from 100% mobile phase A (0.1% FA) to 34% phase B (0.1% FA, 95% ACN), 34-95% at a constant flow rate of 250 nL/min. The mass spectrometer was operated in positive ion mode with data-dependent acquisition. The full scan was acquired in the Orbitrap at 60,000 FWHM resolution in the 400-1600 m/z mass range. Peptide ions were fragmented with CID using a normalized collision energy of 35. Data-dependent acquisition was used to select the 20 most abundant precursor ions for fragmentation. Raw data were accessed in Xcalibur software (Thermo Scientific).

### Database searches and bioinformatics analyses

Raw data were processed using MaxQuant software version 1.5.2.8 (http://www.maxquant.org) and the embedded database search engine Andromeda [PMID: 19029910]. The MS/MS spectra were searched against the UniProt Human (revised) protein database (https://www.uniprot.org), with the addition of common contaminants, with an MS accuracy of 4.5 ppm and 0.5 Da for MS/MS. Cysteine carbamidomethylation (57.021 Da) was set as the fixed modification, and two missed cleavages were set for trypsin. Methionine oxidation (15.994 Da), protein N-terminal acetylation (42.010 Da), and asparagine and glutamine deamidation (+0.984 Da) were set as variable modifications. Proteins were accepted at a false discovery rate (FDR) less than 1%. Proteins with at least two peptides and two ratio counts were accepted for further validation. Label-free quantification was performed using MaxQuant software with the “match between run” feature activated. Statistical analyses were performed on the Perseus version.1.6.10.43 platform (https://maxquant.net/perseus/). Contaminants and proteins identified in the reverse database were excluded before the statistical analyses. Analyses were filtered to include peptides identified and quantified in 2 replicates in at least one condition.

Enriched gene ontology (GO) terms (biological processes and molecular functions) for statistically regulated proteins found in the SphC secretome were analyzed using Panther (https://pantherdb.org). Enriched molecular pathways were analyzed by KEGG (https://www.genome.jp/kegg), Reactome (https://reactome.org), and WikiPathways (https://www.wikipathways.org) platforms at a threshold of q-value <0.05 corrected by Benjamini-Hochberg FDR. Tissue expression analysis was performed using the Human Protein Atlas database (https://www.proteinatlas.org).

### Statistical analysis

Data were first analyzed for Gaussian distribution. Differences among groups were compared using two-tailed, nonpaired Student's *t*-test or ANOVA followed by Tukey's post-test. Analyses were performed using GraphPad Prism 5 software (GraphPad Software, Inc., USA). Differences were considered significant at P<0.05.

### Ethics approval and consent to participate

Mouse studies were approved by the Institutional Care and Use Committee (Comissão de Ética em Uso da Animais do Instituto de Quimica, Universidade de São Paulo (CEUA) No. 42/2016). All methods were carried out in accordance with relevant guidelines and regulations. All methods are reported in accordance with the ARRIVE guidelines.

Human samples were obtained with informed consent according to the Ethics Committee. All methods were carried out in accordance with relevant guidelines and regulations. All experimental protocols were approved by an assigned institutional and/or licensing Committee. The study protocol was approved by Comitê de Ética em Pesquisa do Hospital Universitário da Universidade de São Paulo (CEP-HU/USP 958/09).

## Results

### Human skin cell culture in hanging drops generates a three-dimensional structure named spheroids

**Figure 1:**
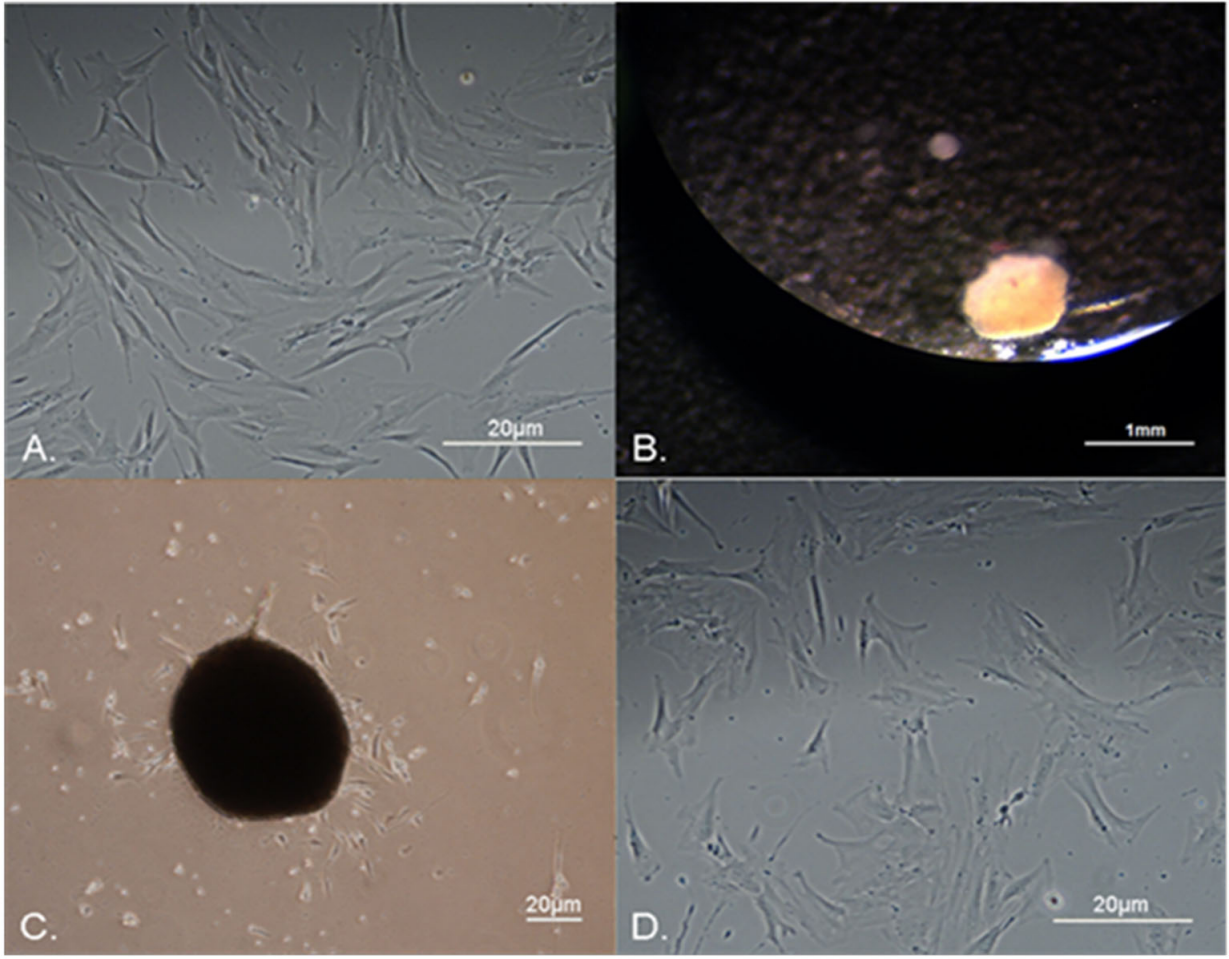
Hanging-drop culture of adult human skin fibroblast-like cells. **A**, Micrograph showing the representative appearance of adult human primary skin cells on adherent plastic, evidencing fibroblast-like forms; scale bar=20 µm. **B**, A representative spheroid of skin-derived cells formed after a hanging drop 72-h culture; scale bar=1 mm. **C**, Cells spontaneously migrate from spheroids that were seeded on an adherent plastic; scale bar=20 µm. **D**, Spheroid-derived cells (SphCs) in adherent plastic culture exhibiting similar morphology to their original primary skin cell counterparts; scale bar=20 µm. Images show representative cultures of cells and spheroids obtained from 3 independent human skin samples. The experiments to obtain spheroids were repeated 6-8 times.

Adult human skin cells in adherent plastic maintained a fibroblast-like morphology (Figure 1A). A total of 5×10^4^ skin fibroblast-like cells at 30 µL/drop resulted in the optimal initial number of cells for spontaneous spheroid generation. Increasing the cell number generated larger spheroids with necrotic cells inside (data not shown). Figure 1B shows a representative compact spheroid with more than 95% of healthy cells (Trypan blue-negative) reaching an average size between 500-700 µm at 72 h. Spheroids were then plated back onto adherent plastic dishes, allowing the spontaneous migration of single cells to form a uniform monolayer (Figure 1C). These cells morphologically resembled the original skin primary cells used for spheroid formation, and we named them spheroid-derived cells (SphCs) (Figure 1D).

### Characterization of SphCs from adult skin fibroblast-like cells

**Figure 2:**
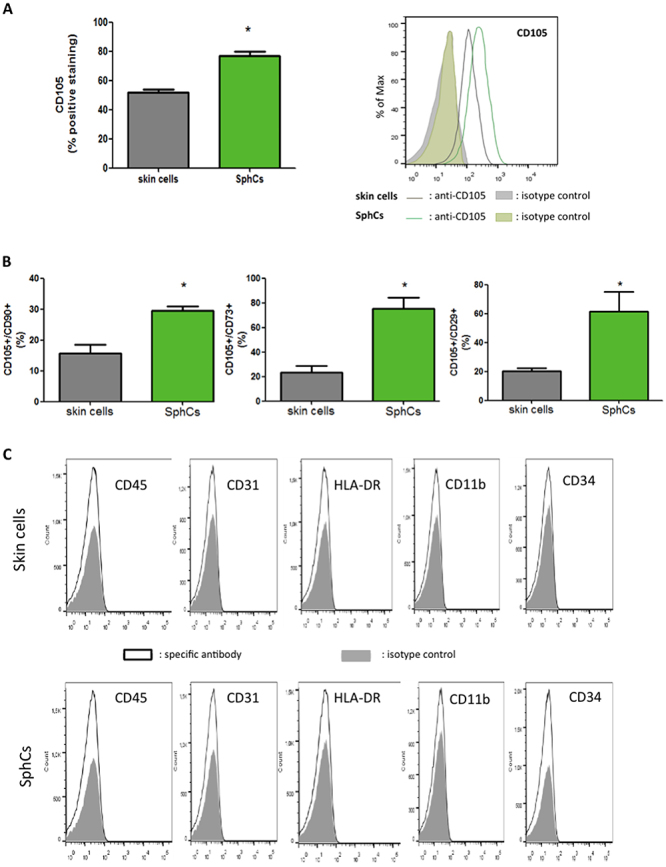
Phenotypic characterization of spheroid-derived cells (SphCs). The surface expression pattern of cluster of differentiation (CD) of SphCs was evaluated in samples from three different patients, and their skin fibroblast-like cell (skin cells) counterparts were used for comparison. **A**, Representative distribution graphs of surface CD105 expression obtained by flow cytometry. The histogram shows the percentage of cells expressing CD105. **B**, Quantification, using flow cytometry, of cells expressing double-positive CD markers as indicated in the figure. **C**, Distribution of skin cells from one donor (upper panel) and SphCs (lower panel) expressing surface human leukocyte antigen-DR (HLA-DR), panleukocyte (CD45), hematopoietic progenitor (CD34), monocyte/macrophage (CD11b), and endothelial (CD31) markers. Similar distribution graphs were obtained with the other two samples. The distribution of CDs in (**A**) and (**C**) is depicted in white, and isotype antibodies are depicted in gray. Data are reported as means±SE; n=3 independent experiments for each skin cell donor and the corresponding SphCs. *P<0.05 *vs* skin cells (*t*-test).

SphCs were immunophenotyped using fluorochrome-conjugated antibodies against CD29, CD73, CD90, and CD105 followed by flow cytometry analysis. The expressions of CD31, CD34, CD45, CD11b, and HLA-DR were also evaluated. SphCs gained surface expression of CD105 (approximately 40% increment) compared with skin parental cells from which they were generated (Figure 2A). Skin cells cultured as spheroids in a single culture showed an increase in double-positive expression of CD105^+^/CD90^+^, CD105^+^/CD73^+^, and CD105^+^/CD29^+^ (Figure 2B).

SphCs and skin fibroblast-like cells expressed undetectable or very low levels of HLA-DR, panleukocyte (CD45), hematopoietic progenitor (CD34), monocyte/macrophage (CD11b), and endothelial (CD31) markers (Figure 2C). Therefore, a single culture step of skin fibroblast-like cells as spheroids conferred characteristic surface expression markers of MSCs to SphCs (5,9).

**Figure 3:**
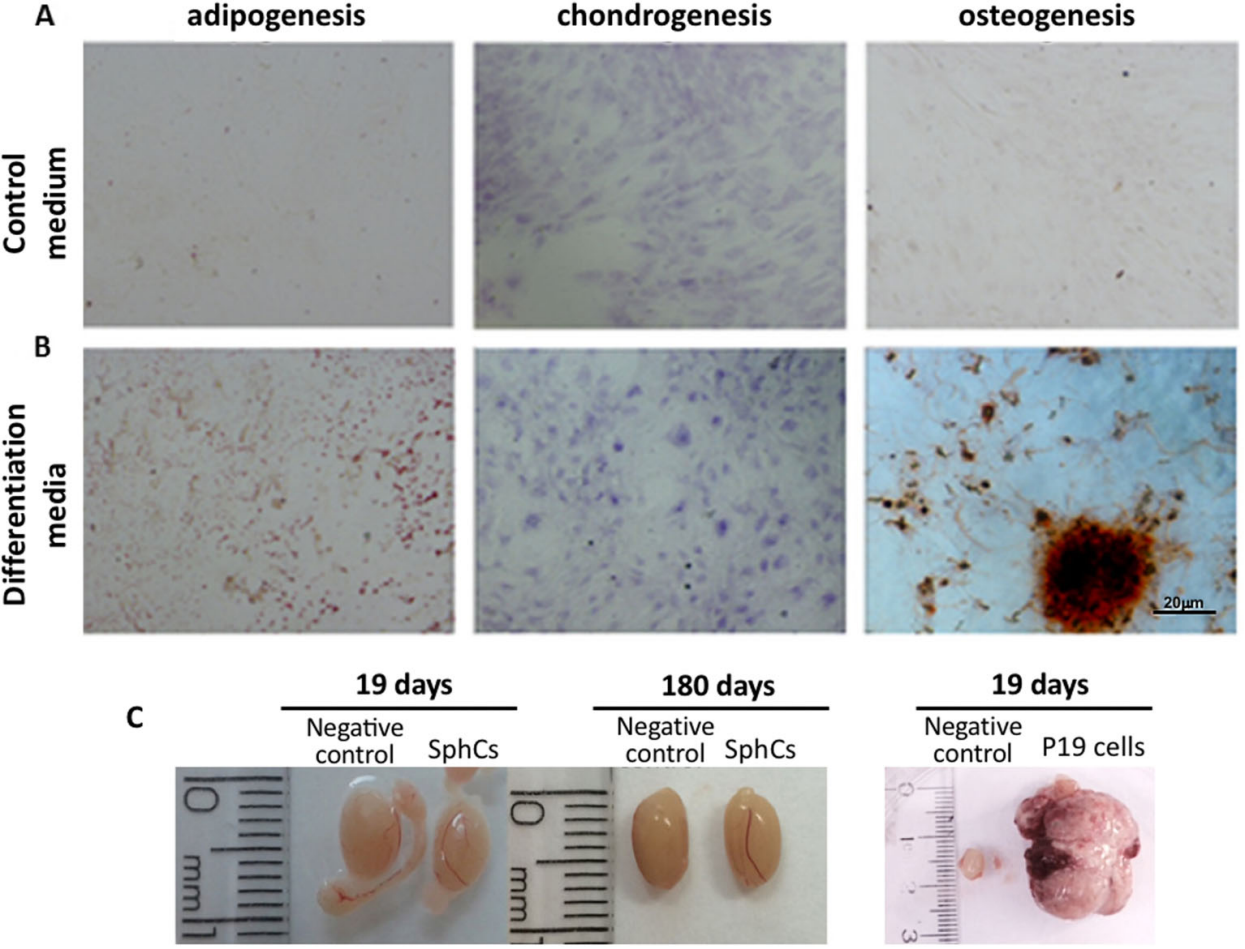
Spheroid-derived cells (SphCs) differentiated into three germ cell lineages. **A**, SphCs grown in adherent plastic with culture medium. **B**, SphCs grown in the presence of tissue-specific differentiation media for 21 days and then fixed and stained with adipocyte- (solution 0.5% Oil Red O in isopropanol, 15 min), chondrocyte- (toluidine blue 0.1%, 30 min), or osteocyte- (2% Alizarin red, 15 min) specific dyes. Representative images (magnification 100×, scale bar 20 μm). The images in (**A**) and (**B**) were obtained from skin cells of one donor. **C**, BALB/c nude mice were subjected to either an intratesticular (*it*) injection of PBS (n=3) as a negative control (left image of each panel) or SphC cell suspension (1×10^5^ in PBS; n=4) in the contralateral testis (middle panel image). The right panel shows a teratoma formation after 19 days post-injection (1×10^5^ P19 cells; n=2) in the right testicle. The images show the testes at 19 and 180 days after *it* injection of SphCs. Testes presented a normal size and morphology until 180 days of observation. Two independent experiments were carried out with cells derived from spheroids from 2 different donors.

SphCs efficiently differentiated into adipocytes, chondrocytes, and osteocytes, as revealed by tissue-specific staining similar to MSCs (Figure 3A and B). Considering SphCs' characteristics, such as growth on adherent plastic, expression of specific surface antigens, and multipotent differentiation potential, they truly meet the minimum criteria to define them as MSC-like cells (9).

To investigate the SphCs potential as therapeutic tools, we evaluated their tumorigenic capacity in a favorable environment, such as that offered by immune-compromised mice. Animals were followed up to 180 days post-administration. Tumorigenic P19 cells generated a tumor 19 days post *it* (the animals were euthanized for humane reasons at this time). SphCs did not form tumors (Figure 3C), nor was any abnormal tissue observed after histochemical evaluation at 180 days, as was seen before (5).

### SphCs downmodulated immune responses *in vitro*

**Figure 4:**
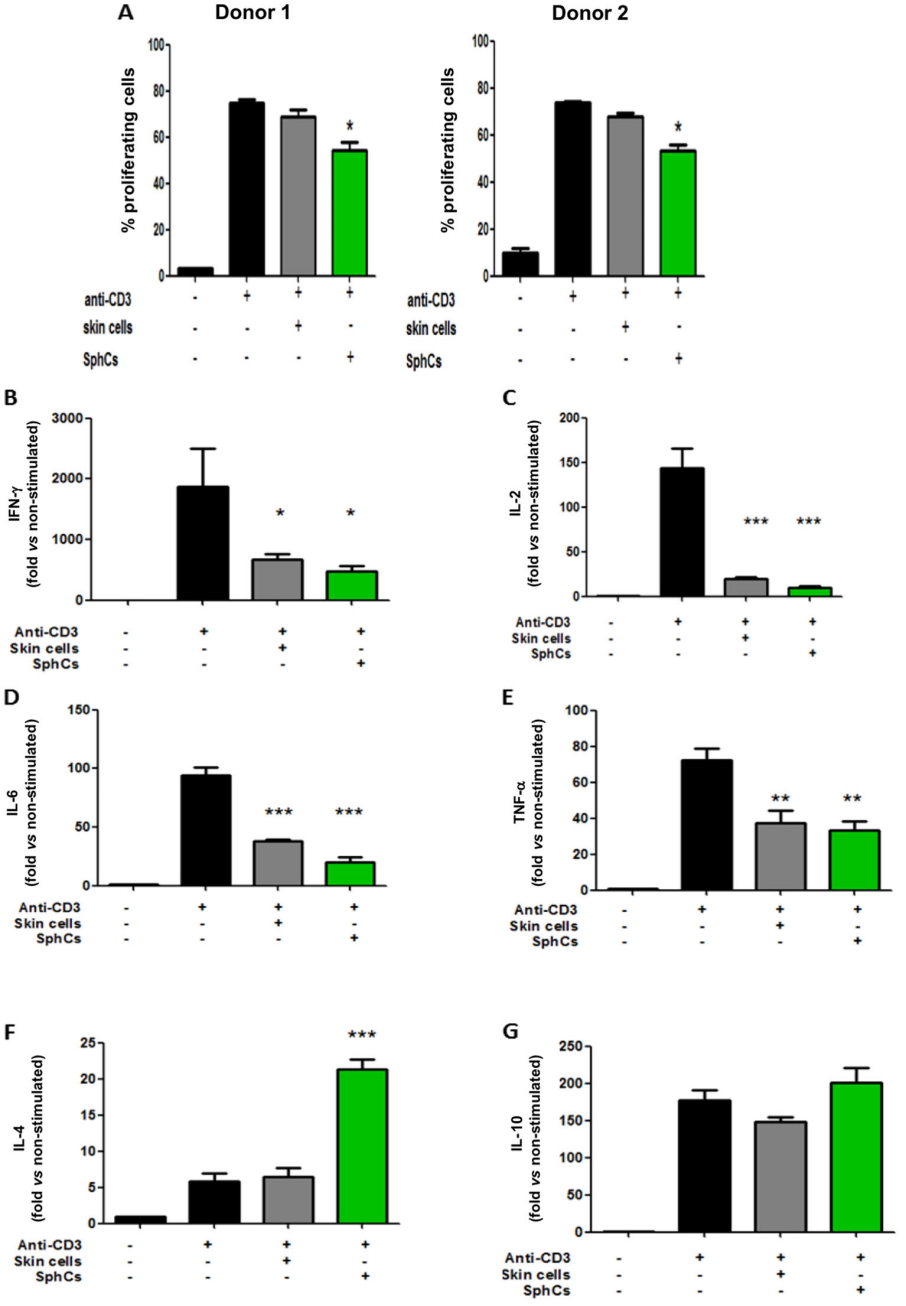
Spheroid-derived cells (SphCs) modulated immune cell responses *in vitro*. **A**, Anti-CD3-stimulated splenocytes (as responder cells; 5×10^5^) were coincubated with SphCs (1×10^4^) or, alternatively, with primary skin cells (1×10^4^). Proliferation was evaluated by CellTrace in T-CD4^+^ lymphocytes by flow cytometry. Assays were performed employing two different donors; n=4 independent experiments. **B**-**G**, Cytokine secretion by activated splenocytes was biased in the presence of SphCs. Splenocytes were activated by an anti-CD3-coated surface. Secreted cytokines were quantified by CBA multiplex assay: **B**, Interferon (IFN)-γ; **C**, Interleukin (IL)-2; **D**, IL-6; **E**, tumor necrosis factor (TNF)-α; **F**, IL-4; **G**, IL-10. *P<0.05, **P<0.01, ***P<0.001 *vs* anti-CD3 stimulated splenocytes (ANOVA). Data are reported as means±SE; n=3 independent experiments.

BALB/c splenocytes were employed to evaluate SphCs immune regulatory capacity. Anti-CD3-stimulated T lymphocyte proliferation was compared under the influence of SphCs or alternatively their skin fibroblast-like counterparts. There was a reduction in T-CD4^+^ lymphocyte proliferation when these cells were cocultured with SphCs, while the presence of skin fibroblast-like cells did not alter proliferation rates (Figure 4A).

We analyzed the profile of cytokines secreted by lymphocytes stimulated with anti-CD3 in the presence of SphCs *in vitro*. Cytokines were quantified by flow cytometry in the 36-h spent-supernatants of anti-CD3-primed splenocytes cocultured with either skin fibroblast-like cells or SphCs. The hallmark Th1 cytokine INF-γ was dramatically downregulated by the presence of both SphCs and skin fibroblast-like cells (Figure 4B). Relevant cytokines required for an effective proinflammatory response, such as IL-2, IL-6, and TNF-α, were less abundant in the supernatant derived from both skin fibroblast-like cells and SphCs than in the positive control (anti-CD3 alone), and the levels were even lower in the presence of SphCs (Figure 4C, D, and E). IL-4 favors the induction of a Th2 lymphocyte profile. SphCs induced a 4-fold increase in the secretion of IL-4 in comparison with anti-CD3 stimulation, while skin fibroblasts did not show any effect (Figure 4F). SphCs did not stimulate secretion of IL-10, a classic immune-modulatory cytokine, after CD3 activation (Figure 4G). There were no differences in the secretion of IL-17A between cocultures and the control (not shown).

Taking into account the proliferation and cytokine profile of T-CD4^+^ lymphocytes, SphCs were capable of inducing a trend toward a decrease in the Th1/Th2 lymphocytes ratio, supporting an anti-inflammatory environment.

### SphC-conditioned media attenuated proinflammatory cytokine-induced islet death

**Figure 5:**
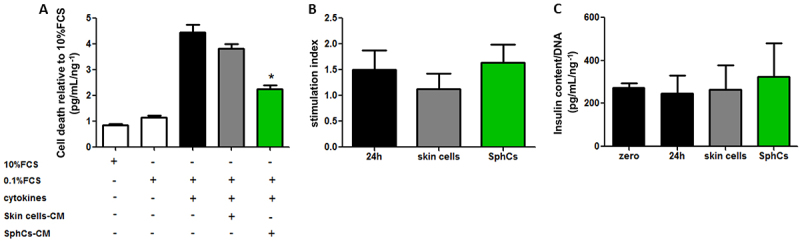
Spheroid-derived cell (SphCs)-conditioned media attenuated cytokine-induced islet cell death without altering insulin secretion. **A**, Cell death in isolated murine islets was evaluated after 24 h of incubation (0.1% FCS) and challenged with a cocktail of proinflammatory cytokines supplemented or not with 20% V/V conditioned media from parental skin cells (skin cell-CM) or, alternatively, SphCs-CM. Data were calculated relative to the negative control condition (10% FCS) and are reported as means±SE; n=4 independent experiments (each experiment included at least 8 islets analyzed per condition). *P<0.05 *vs* cytokines and skin cell-derived CM. **B**, Glucose-stimulated insulin secretion (stimulation index) of islets under 24 h of CM from skin cells or SphCs. The stimulation index was calculated as follows: (secreted insulin concentration in 16.7 mM glucose buffer) / (secreted insulin concentration in 2.8 mm glucose buffer). Data are reported as means±SE; n=3 independent experiments. **C**, Total insulin content in murine islets under 24 h of CM (20% V/V) from skin cells or SphCs. Insulin was measured by ELISA and normalized to DNA content. Data are reported as means±SE; n=3 independent experiments. ANOVA was used for statistical analyses.

Islets cultured with a cocktail of proinflammatory cytokines, mimicking the unfavorable microenvironment to which they are exposed during the autoimmune process of T1D, died by apoptosis. We assessed whether soluble factors secreted by SphCs might protect isolated islets from the proinflammatory damage exerted by cytokines. After 24 h, the cocktail of proinflammatory cytokines induced a 4-fold increase in islet death relative to the control (islets in optimal conditions, e.g., 10% FBS). SphCs supernatants containing soluble factors reduced islet death compared with proinflammatory cytokine stimuli (approximately 50% reduction). There was a lack of beneficial effect on islet survival when employing conditioned media from skin fibroblast-like cells (Figure 5A).

Conditioned media from both SphCs and skin fibroblast-like cells did not perturb glucose-stimulated insulin secretion or total islet-insulin content, indicating that soluble factor/s would only affect islet survival (Figure 5B and C).

### Cotransplantation of marginal mass allogeneic islets with SphCs improved glycemic control in diabetic mice

**Figure 6:**
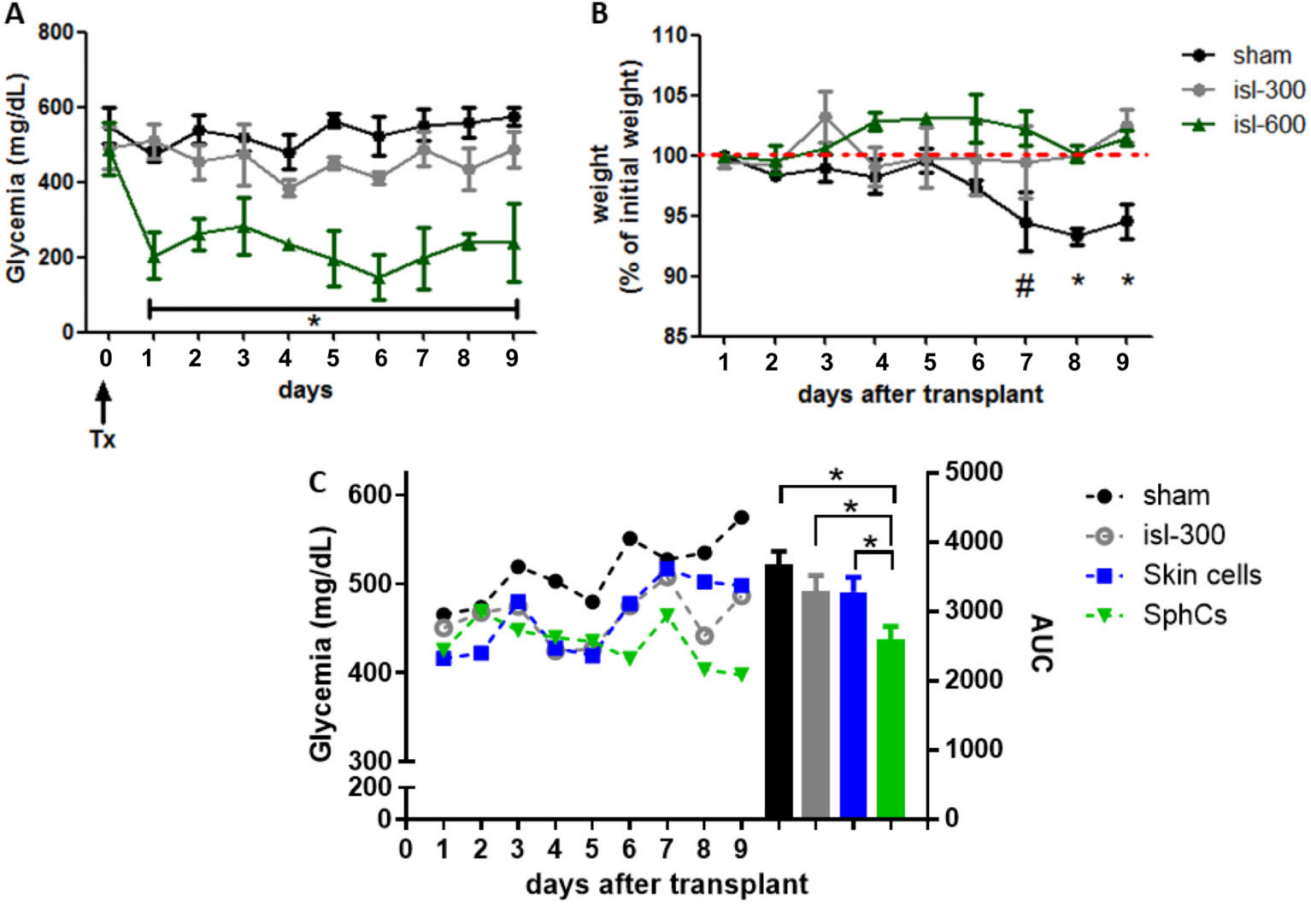
Cotransplantation of marginal mass allogeneic islets with skin spheroid-derived cells (SphCs) improved glycemic control in diabetic mice. **A**, Establishment of the marginal mass allogenic islets. Both 300 islet equivalents (IEQ)-transplanted and sham-transplanted diabetic mice showed no difference in blood glucose. Transplantation of islets at a dose of 600 IEQ effectively restored normoglycemia. Data are reported as means±SE (n=3 mice per experimental group). *P<0.05 *vs* sham and isl-300. **B**, Both 300 IEQ and 600 IEQ recipient diabetic mice did not lose body weight. On the contrary, sham-operated mice showed the onset of body weight loss starting from day 6 post-surgery. Data are reported as means±SE (n=3 mice per experimental group). ^#^P<0.05 *vs* isl-600; *P<0.05 *vs* isl-300 and isl-600. **C**, Cotransplantation of marginal mass allogenic islets with SphCs (1×10^4^) improved glycemia in diabetic mice. The results are shown as plasma glucose concentration in the animals at different time points (left Y axis) and by integrating the area under the curve (AUC) as depicted in the right “Y” axis. Data are reported as means±SE (n=9 mice per experimental group) *P<0.05 *vs* SphCs. ANOVA was used for statistical analyses.

We established the marginal mass of allogeneic islets using 300 IEQ transplanted under the kidney capsule of diabetic C57BL/6 mice. Transplantation of 300 IEQ maintained glycemic levels slightly below those of sham-operated mice (Figure 6A). As a control, engraftment of 600 IEQ from BALB/c mice into C57BL/6 diabetic mice dramatically decreased glycemia as soon as 1 day post-administration. Figure 6B shows animal weights throughout the experimental period. The marginal mass of allogeneic islets had a comparable effect on maintaining body weight to transplantation of 600IEQ, while diabetic surgical sham animals experienced significant weight loss (Figure 6B). As expected, the transplantation of allogeneic islets at a dose of 300 IEQ or the same number of islets cotransplanted with skin cells resulted in a slight reduction in hyperglycemia. However, the cotransplantation of the marginal mass of allogeneic islets with SphCs (10^4^) led to significant improvement in glycemic control compared to the sham animals (Figure 6C).

### SphCs protein secretome analysis

Having established that conditioned media of SphCs both immunomodulated T-CD4^+^ lymphocyte responses and exerted cytoprotection on cytokine-challenged islets, we analyzed the protein secretome of SphCs and primary skin fibroblast-like cells using a comparative proteomic approach. After comparing the abundance of proteins present in both secretomes, we classified protein components identified in three different groups according to the ratio of abundance in the secretome of SphCs versus the one obtained in the corresponding primary skin culture. We established a cutoff value ≥1.5 or ≤0.65 for more abundant and less abundant proteins, respectively. Supplementary Tables S1 and S2 summarize the protein components of SphCs presenting an increase (upregulated) or decrease (downregulated) in relative abundance compared with the corresponding skin fibroblast-like cells. Approximately 33% of the proteins were upregulated in the SphC secretome, while almost 46% of them were downregulated.

**Figure 7:**
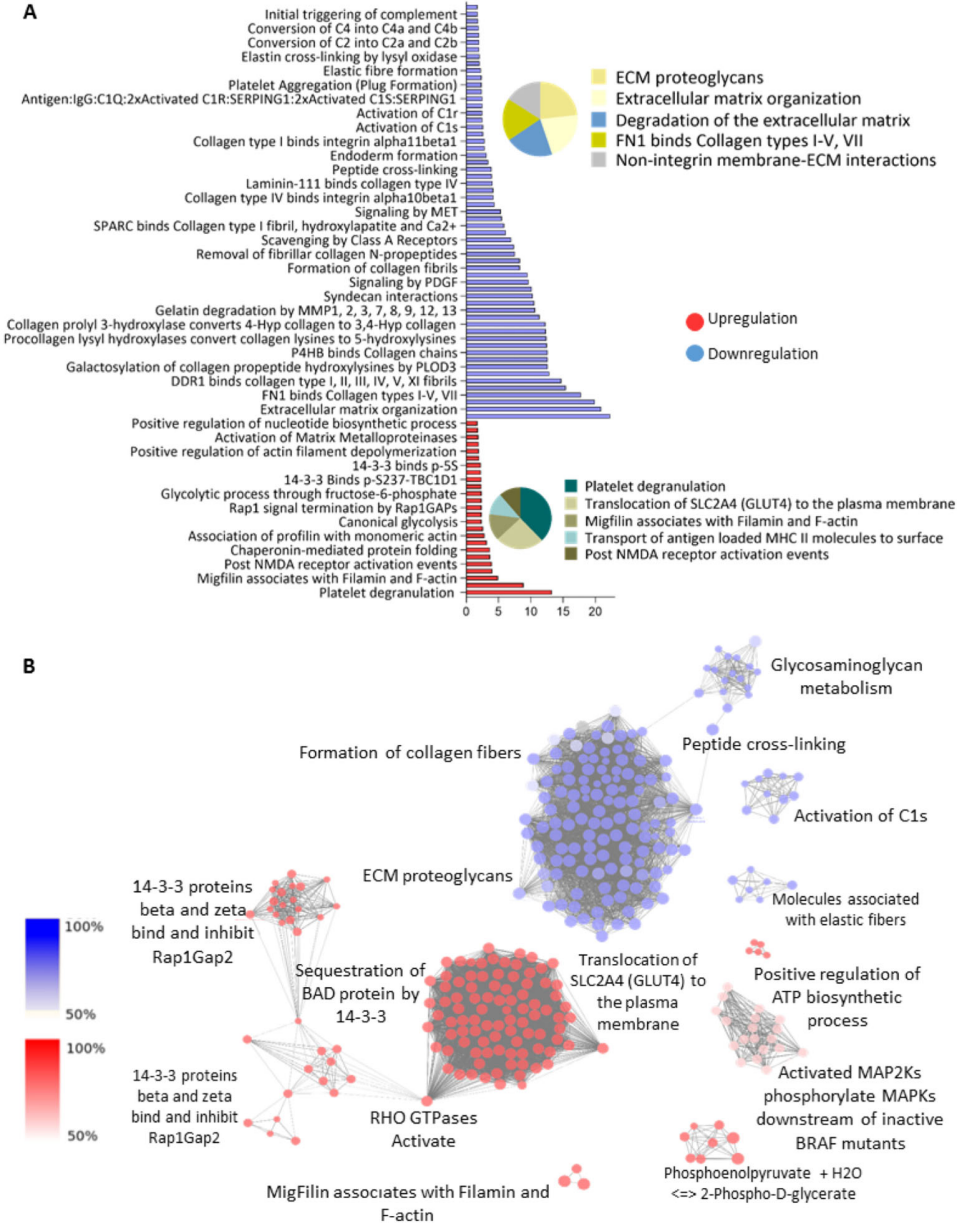
Protein secretome analysis of skin spheroid-derived cells (SphCs) compared to that obtained in primary cultures of skin cells. **A**, Biological processes and enriched molecular pathways associated with differentially regulated proteins (q-value <0.05, Bonferroni, ClueGO app) in the secretome of SphCs compared with skin fibroblast-like cells. The colors red and blue indicate upregulated and downregulated ontologies, respectively. **B**, Cluster networks of protein-protein interactions corresponding to the main processes described in (**A**) were analyzed by the STRING database. A minimum required interaction score of 0.7 was used to build a high confidence interaction network. The line connecting the proteins, depicted as spheres, shows experimental evidence of interaction between them. Each network presents a PPI enrichment value <1exp-6.

Additionally, each set of identified proteins was submitted to interactome analysis to understand their putative cellular functions (Figure 7, Supplementary Tables S3 and S4). Among the underexpressed proteins, we identified proteins related to extracellular matrix remodeling (e.g., actin, laminin, tubulin, collagen, elastin, lumican, SPARC, and thrombospondin-1), collagen formation, complement C1-related proteins, and others associated with elastic fibers (elastin and other proteins), and glycosaminoglycan metabolism (which have functions in the regulation of cell growth, proliferation, promotion of cell adhesion, and anticoagulation).

Among the upregulated proteins, there were Rho GTPases that coordinately activate several transduction pathways, proteins involved in glucose uptake and its metabolism (GLUT4, enolases), regulation of apoptosis (e.g., 14-3-3), and other mechanisms involved in cellular development, cell survival (e.g., gremlin), and stress response (pentraxin-3), and migfilin, which regulates remodeling, morphology, and motility processes.

In summary, this protein secretome analysis highlighted the expression pattern of proteins secreted by SphCs that might be responsible for islet survival, acceleration of glucose metabolism, and tissue repair.

## Discussion

In T1D, most of the β cells are destroyed and some may remain dedifferentiated (10). Administration of insulin often fails to maintain precise glycemic control, and transplantation of a solid pancreas or isolated islets from cadaveric donors are options to achieve insulin independence and/or reduce fluctuations in blood sugar levels. Pancreas transplantation is considered invasive and is often associated with comorbidities (1). Therefore, islet transplantation is an appealing therapeutic option for labile T1D patients. Efforts in clinical research have contributed significantly to improving islet transplantation, achieving exogenous insulin independence in 50% of patients after 5 years of multidonor infusions (11).

Preservation of islet viability remains challenging because of a significant loss of functionality due to both mechanical and oxidative stress as well as activation of apoptotic pathways (12). Novel procedures to maintain islet viability and functionality before and after infusion can enhance transplantation outcomes.

Cotransplantation of stem cells with isolated islets has been investigated as a translational option for T1D. MSCs have been noted because of their anti-inflammatory, angiogenic, and immunoregulatory capacities, and as a source of nourishing factors for islets (13). Use of MSCs has improved experimental diabetes using syngeneic and allogeneic settings and in immune-deficient mice (6,7,9). MSCs repair or attenuate tissue destruction through paracrine secretions or cell-to-cell contacts modulating inflammatory and immune responses (8,14,15). MSCs cultured as spheroids increase the surface expression of CXCR4 and adhesion to endothelial cells (16).

Because obtaining human skin tissue is safe and minimally invasive, we evaluated whether skin fibroblast-like cells after aggregation in culture could contribute to improving the outcomes of islet transplantation in diabetic mice. Our findings underscore the potential benefits of cotransplantation of SphCs with marginal mass allogeneic islets, which could be attributed to their role in promoting islet engraftment and indirectly attenuating the host's alloimmune response. The observed overall increase in islet cell viability led to better glycemic control in diabetic transplanted mice. Aggregation promotes cell-to-cell contacts that may help maintain the stem cell properties of SphCs, as evidenced in previous studies using 3D culture of MSCs (17).

Migrating cells from spheroids, e.g. SphCs, differentiated after induction in adipocytes, osteocytes, and chondrocytes, similar to conventional MSCs. We found no expression of HLA-DR on the surface of SphCs, and they may remain undetectable to allogeneic effector T-CD4^+^ lymphocytes. After several months of SphCs administration, SphCs did not show proliferation, differentiation, or teratoma formation in immunodeficient mice, demonstrating their safety and potential therapeutic utility.

SphCs share immunoregulatory traits with MSCs. Our study provided novel findings on how SphCs can effectively reduce T-CD4^+^ cell proliferation and modulate cytokine profiles of stimulated splenocytes *in vitro*, favoring an increase in the Th2/Th1 ratio.

We described potent immunomodulatory features of a unique human stem cell population (Muse) derived from adipose tissue (5,6). Both Muse cells and ShpCs grow in suspension as spheroids. Therefore, aggregation could be a driving force for cells to express cytokines and factors, such as TGF-β1 released by Muse cells, as mediators of their immunomodulatory actions. Noteworthy, a single culture step as a spheroid is sufficient to acquire such properties.

CD73 has ecto-5'-nucleotidase activity influencing immune responses such as inducing immune tolerance, T lymphocyte differentiation, and tilting the balance toward immune-suppressive microenvironments (18,19). SphCs gained surface expression of CD73. The pronounced nucleotidase activity exhibited on the surface of SphCs could potentially elevate nucleoside levels, leading to the inhibition of proliferation and activation of allogeneic effector T lymphocytes (19). Antigen-specific T lymphocytes are involved in the autoimmune process that destroys β cells in T1D. The early stages of the disease mobilize inflammatory infiltration in the islets (insulitis) (20), contributing to β cell dysfunction and destruction (21). Cytokines secreted by infiltrated immune cells such as IL-1β, IFN-γ, and TNF-α impair the function and viability of β cells (22). Interestingly, SphCs-conditioned media promoted inhibition of islet cell death when challenged by a mixture of proinflammatory cytokines *in vitro*. Growth factors secreted by MSCs (23,24) may conserve islet integrity, growth, and functionality. The mechanisms underlying the protective effects of SphCs on β cells remain elusive. However, it is hypothesized that they involve the modulation of key regulators of β cell proliferation, such as Akt and Erk (25). The potential impact of SphCs-conditioned media on cell proliferation and regeneration of β cells may require longer culture periods of islets. Likewise, we did not observe improvements in basal or glucose-stimulated insulin secretion in islets exposed to SphCs-conditioned media.

Transplanted islets encounter a rapid attack mediated by allogeneic destructive reactions after infusion (26,27). The addition of MSCs at the site of transplantation may improve immune tolerance and protect islets, avoiding the need for administration of toxic immunosuppressive drugs. Our study revealed that cotransplantation of marginal mass allogeneic islets with SphCs resulted in enhanced glycemic control in STZ-induced diabetic mice. We speculate that this improvement was due, in part, to the release of pro-survival factors by SphCs that positively influence the islets, along with the creation of a favorable anti-inflammatory microenvironment. In addition, SphCs also exhibit immune-regulatory activity, which helps in controlling alloreactive T lymphocytes.

The partial restoration of glycemia in diabetic mice upon cotransplantation of marginal mass allogeneic islets with SphCs without reaching normal glucose levels could be attributed to the use of a relatively low number (10^4^ cells) of administered SphCs. Similar experiments employing a mixture of MSCs and islet-derived single cells at a 1:1 ratio transplanted in a mouse model of T1D reached lasting normoglycemia (28). Considering that the estimated number of cells in an islet is ∼1500 (150 µm diameter) (29), we cotransplanted SphCs:Islet-cells at a 1:45 ratio. In future experiments, increasing this ratio by at least ten-fold may potentially lead to improved outcomes.

Proteomics studies have provided valuable insights into the distinct protein profiles of SphC-secreted proteins compared to those of skin cells. We identified numerous biological processes and intracellular pathways that are differentially regulated between SphCs and skin fibroblast-like cell-conditioned media. We identified proteins associated with β cell cytoprotection and modulating immune responses. The top hits on the functions displayed by these proteins were related to extracellular matrix remodeling, modulation of apoptosis, and cell differentiation. Low levels of matrix metalloproteases might lead to an increased activity of membrane-bound FASL.

As a result, the enhanced apoptotic capability of SphCs expressing FASL on effector T cells while leaving Treg cells unaffected would likely lead to improved β cell viability and increased lifespan of islet graft (30).

We found upregulated metalloproteases inhibitors in the SphCs secretome that could, partially, contribute to the less aggressive proinflammatory microenvironment for islets. The 14-3-3ζ involved in the inhibition of β cell apoptosis (31) was upregulated in the SphCs secretome. Both g remlin-1 and pentatraxin-3, also abundant in the secretome of SphCs, have been related to growth and survival in response to inflammation and tissue damage (32,33). In tubular epithelial cells, gremlin-1 increased TFG-β production through Smad activation, inducing a myofibroblast-like phenotype (34).

This work describes the use of transplanted SphC to establish a conducive microenvironment for alloislet engraftment, thereby preserving their viability and functionality without the need for immunosuppressive drugs. Moreover, it provides evidence of the secretion of bioactive factors by SphCs that may enhance the survival of β cells in allogeneic settings. Further elucidation and characterization of these factors could offer a promising alternative to cell-based approaches, potentially paving the way for novel therapeutic interventions.

Continued advancements in generating more efficient SphCs can improve the field of islet transplantation, bringing us closer to achieving insulin independence for patients. By endowing β cells with enhanced cytoprotection through SphCs, it may be possible to circumvent the need for chemotherapy-induced immune suppression, opening new avenues for safer and more effective treatments. This knowledge can alleviate organ shortage by providing an alternative approach.

## Supplementary Material

Click to view [pdf].
